# Secretory phospholipase A2 in SARS-CoV-2 infection and multisystem inflammatory syndrome in children (MIS-C)

**DOI:** 10.1177/15353702211028560

**Published:** 2021-07-13

**Authors:** Frans A Kuypers, Christina A Rostad, Evan J Anderson, Ann Chahroudi, Preeti Jaggi, Jens Wrammert, Grace Mantus, Rajit Basu, Frank Harris, Bradley Hanberry, Andres Camacho-Gonzalez, Shaminy Manoranjithan, Miriam Vos, Lou Ann Brown, Claudia R Morris

**Affiliations:** 1 Division of Hematology, Department of Pediatrics, University of California, San Francisco, CA 94609, USA; 2 Department of Pediatrics 1371 , School of Medicine, Emory University, Atlanta, GA 30322, USA; 3 Children’s Healthcare of Atlanta, Atlanta, GA 30322, USA; 4 Center for Childhood Infections and Vaccines of Children’s Healthcare of Atlanta and Emory University, Atlanta, GA 30322, USA; 5 Department of Medicine, School of Medicine, Emory University, Atlanta, GA 30322, USA; 6 Center for Clinical and Translational Research, Children’s Healthcare of Atlanta and Emory University, Atlanta, GA 30322, USA

**Keywords:** SARS-CoVC-2, COVID-19, secretory phospholipase A2, multisystem inflammatory syndrome in children

## Abstract

Secretory phospholipase 2 (sPLA2) acts as a mediator between proximal and distal events of the inflammatory cascade. Its role in SARS-CoV-2 infection is unknown, but could contribute to COVID-19 inflammasome activation and cellular damage. We present the first report of plasma sPLA2 levels in adults and children with COVID-19 compared with controls. Currently asymptomatic adults with a history of recent COVID-19 infection (≥4 weeks before) identified by SARS-CoV-2 IgG antibodies had sPLA2 levels similar to those who were seronegative (9 ± 6 vs.17 ± 28 ng/mL, *P* = 0.26). In contrast, children hospitalized with severe COVID-19 had significantly elevated sPLA2 compared with those with mild or asymptomatic SARS-CoV-2 infection (269 ± 137 vs. 2 ± 3 ng/mL, *P* = 0.01). Among children hospitalized with multisystem inflammatory syndrome in children (MIS-C), all had severe disease requiring pediatric intensive care unit (PICU) admission. sPLA2 levels were significantly higher in those with acute illness <10 days versus convalescent disease ≥10 days (540 ± 510 vs. 2 ± 1, *P* = 0.04). Thus, sPLA2 levels correlated with COVID-19 severity and acute MIS-C in children, implicating a role in inflammasome activation and disease pathogenesis. sPLA2 may be a useful biomarker to stratify risk and guide patient management for children with acute COVID-19 and MIS-C. Therapeutic compounds targeting sPLA2 and inflammasome activation warrant consideration.

## Impact statement

sPLA2 acts as a mediator between proximal and distal events of the inflammatory cascade, and we report for the first time that sPLA2 is elevated in children with acute COVID-19 infection and MIS-C. sPLA2 correlated with pediatric COVID-19 severity and acute MIS-C, implicating a potential role in inflammasome activation and disease pathogenesis. This study helps fill the void of mechanistic data in the literature on etiology of inflammation in pediatric COVID-19 infection and MIS-C. The value of sPLA2 as a biomarker of inflammation and potential therapeutic target warrants investigation; this work may lead to novel interventions for COVID-19 infection and MIS-C.

## Introduction

SARS-CoV-2 is currently causing a devastating pandemic and there is a pressing need to understand the mechanisms of disease in order to rapidly develop novel therapeutics. Systemic inflammation is a component of severe disease in the clinical spectrum of COVID-19.^1^

Phospholipases are a large family of enzymes that facilitate the degradation of lipids.

The group II secretory phospholipase A2 (sPLA2) is an important constituent of an interactive network of enzymes, lipid mediators, and cytokines which contribute to normal physiology as well as pathophysiology.^2^ The lipolytic activity of sPLA2 releases fatty acids from the sn-2 position of membrane phospholipids, ultimately generating important lipid mediators such as prostaglandins, leukotrienes, and platelet activating factor.^3^ Arachidonic acid, and its numerous metabolites, act as intracellular and intercellular messengers contributing to normal cell physiology by modulating enzyme activities and ion channels. The products of sPLA2 are substrates for inflammatory lipid mediators that play important roles in the pathogenesis of inflammatory diseases.^2^ Formation of sPLA2 and C-reactive protein (CRP) is initiated by proinflammatory cytokines (IL-1, IL-6, TNF-α) under control of glucocorticoids.^4^ sPLA2 acts as a mediator between proximal and distal events of the inflammatory cascade. Upon stimulation, an increase of mRNA levels for sPLA2 is observed in several different tissues including renal mesenchymal cells, chondrocytes, vascular smooth muscle, osteoblasts, and endothelial cells. An increase in enzyme activity paralleled by an increase in concentration has been found in human disease including rheumatoid arthritis, septic shock, acute myocardial infarction, Crohn’s disease, hematological malignant disorders, febrile bacterial infections, ulcerative colitis, and nephropathy,^2^ and often correlates with disease severity.^5^ sPLA2 is also well known to be involved in lung inflammation and surfactant degradation based on animal and human studies,^6,7^ which may be relevant to COVID-19 infection. In contrast to the closely related phospholipases from snake and bee venom, sPLA2 will not randomly break down normal human cell membranes, but it will attack bacterial membranes or apoptotic cells that expose phosphatidyl serine (PS).^8^ We have previously shown that the level of sPLA2 measured in patients with sickle cell disease (SCD) is a harbinger of the onset of acute chest syndrome (ACS),^9,10^ and predicts severity of cellular damage in trauma.^11^ We have also observed very high sPLA2 levels in two children diagnosed with Kawasaki disease (KD), early in the course of their illness.^12^

Despite its important role in inflammatory processes, sPLA2 has not yet been studied in COVID-19, the disease caused by SARS-CoV-2. COVID-19 severity has been reported to be associated with elevated levels of CRP,^13^ apoptosis,^14–16^ and related cell damage;^17^ however, knowledge of the correlations between several biomarkers and COVID-19 is limited, and the pathogenesis of multiorgan damage is unclear.^18^ We hypothesized that sPLA2 plasma levels in patients with COVID-19 could correlate with disease severity. In this study, we evaluated this premise in a cohort of pediatric patients with acute COVID-19 and those with multisystem inflammatory syndrome in children (MIS-C) associated with COVID-19, which shares features with KD.^19–26^

## Materials and methods

### Subjects

This was a prospective observational study involving two patient cohorts. The first was a case–control study of asymptomatic pediatric health-care workers (HCWs) ≥18 years of age, screened between April and June 2020 for SARS-CoV-2 IgG antibodies^27^ as part of a longitudinal COVID-19 surveillance study.^28^ Those who tested positive for SARS-CoV-2 IgG antibodies were matched by age and gender with HCWs who tested negative for IgG antibodies. The second observational cohort included children 0–21 years of age hospitalized at Children’s Healthcare of Atlanta (CHOA) between March and May 2020 with asymptomatic SARS-CoV-2 (identified through screening by PCR), confirmed or suspected COVID-19, MIS-C, or KD who were enrolled for prospective and/or residual blood collection and had plasma samples available for analysis. Cohorts were defined as having COVID-19 if they tested positive for SARS-CoV-2 by nasopharyngeal (NP) PCR and had symptoms consistent with COVID-19; MIS-C if they tested positive for SARS-CoV-2 by either NP PCR or commercial IgG antibody test (Abbott), and met the case definition proposed by the Centers for Disease Control and Prevention (CDC; https://emergency.cdc.gov/han/ 15 May 2020) and KD if they met the American Heart Association diagnostic criteria for complete or incomplete KD.^29^ Hospitalized controls were defined as patients evaluated for any of the above conditions, but did not meet diagnostic criteria. A convenience sample of asymptomatic patients testing positive for SARS-CoV-2 by PCR, but hospitalized for other reasons requiring screening preoperatively or for intensive care unit admission was also included. Serologic data for a subset of this cohort has been previously published; however, the cohorts are not identical due to plasma sample availability and different enrollment periods.^30^ Demographic information was collected from both the adult and pediatric cohorts. Clinical course and laboratory data were extracted from the electronic medical record of the pediatric cohort.

### Measurements

Patient blood was collected in EDTA or CPT tubes, and plasma was separated by centrifugation and stored at −20°C until analysis. Both the HCW cohort and the pediatric cohort were tested for SARS-CoV-2 antibodies by measuring the IgG antibody responses to the receptor binding domain of the spike protein using an enzyme-linked immunosorbent assay (ELISA) as previously described.^27^ The pediatric cohort was also tested for SARS-CoV-2 using clinician-ordered tests, which included SARS-CoV-2 NP PCRs and/or nucleocapsid IgG antibody ELISAs (Abbott). sPLA2 was measured with an ELISA kit (Cayman Chemical, Ann Arbor, MI) using the manufacturers’ provided protocol. In the HCW cohort, CRP (Cayman Chemical) and F1.2 (MyBioSource Inc., San Diego, CA) were also measured using ELISA kits and the manufacturers’ protocols.

For the asymptomatic adult HCW cohort, sPLA2 levels were measured in patients free of symptoms for greater than two weeks. For the pediatric cohort, sPLA2 levels were measured in all available samples and were compared among four groups (SARS-CoV-2 positive by PCR, MIS-C, KD, and hospitalized controls). Because some children identified with SARS-CoV-2 lacked symptoms of COVID-19, but were hospitalized for other conditions for which screening for SARS-CoV-2 by PCR was performed (e.g. Tylenol ingestion, appendicitis, presyncope with underlying congenital heart disease), we performed a subgroup analysis of children hospitalized with symptomatic COVID-19 versus children who had mild or asymptomatic SARS-CoV-2 infection that were ostensibly hospitalized for other reasons. Samples from children with MIS-C that were obtained early in the disease process (<10 days following symptom onset) were compared with later samples (≥10 days following symptom onset) in a subgroup analysis.

### Data analysis

Demographic data were reported as frequencies for categorical data and medians for ordered normal data. Means (± standard deviations) were used for continuous normal data, while the Pearson correlation coefficient was used to report correlations. Statistical significance (*P* value) was reported using two-tailed unpaired Student’s *t*-test for continuous data, Fischer’s exact test was used for categorical data, and Mann-Whitney U test was used for ordered data in the adult HCW cohort (alpha = 0.05). One-way ANOVA was used for continuous data and Chi-squared test was used for categorical data in the pediatric patients hospitalized with illness (alpha = 0.05).

### Study approval

These studies received Institutional Review Board approval from Emory University; the pediatric study was also reviewed and approved by CHOA. Electronic informed consent was received from HCW participants prior to inclusion in the study, while informed consent was obtained from all parents/guardians and age-appropriate assent was obtained for prospective enrollment into the pediatric study. Residual samples were collected through waiver of informed consent.

## Results

### Adult health-care worker cohort

Participant demographics and laboratory values for the healthy adult health care worker (HCW) cohort are summarized in Table 1. Fourteen healthy SARS-CoV-2 IgG-negative HCWs were matched by age and gender to 14 seropositive HCWs. No IgG-positive HCWs required hospitalization, and all had been asymptomatic for >2 weeks prior to enrollment. The mean time from onset of viral symptoms in the seropositive group reporting viral illness (8/14, 57%) was 58 ± 31 days with a range of 29–120 days. Samples therefore represented convalescent titers. Only 3/14 (21%) seropositive HCWs had a history of a positive SARS-CoV-2 PCR performed for clinical evaluation during acute illness; 6/14 (43%) had been completely asymptomatic since January 2020. No statistically significant difference in sPLA2 was observed between the seropositive versus seronegative groups. There was no correlation identified between sPLA2 levels and IgG titers in seropositive patients. The prothrombinase fragment 1.2 (F1.2) levels and CRP were also similar between groups.

**Table 1.** Healthcare Worker Cohort demographics and laboratory values.

Variables	All (*N* = 28)	Seropositive (*N* = 14)	Seronegative (*N* = 14)	*P value*
Age range years, median	41–50	41–50	41–50	1
Gender, male (%)	29%	29%	29%	1
Laboratory values				
CRP (µg/mL)	1.5 ± 1.8	2.0 ± 2.7	1.1 ± 0.7	0.33
F1 + 2 (pmol/mL)	0.45 ± 0.13	0.44 ± 0.16	0.45 ± 0.12	0.77
sPLA2 (ng/mL)	13 ± 21	9 ± 6	17 ± 28	0.26

CRP: C-reactive protein; F1.2: fragment 1.2; sPLA2: secretory phospholipase 2.

Mann-Whitney U test, Fischer’s exact test, or unpaired Student’s *t*-test; alpha = 0.05. Data are represented as median for ordinal data, *N* (%) for nominal data or mean ± SD for continuous data.

### Hospitalized pediatric cohort

**Figure 1.**
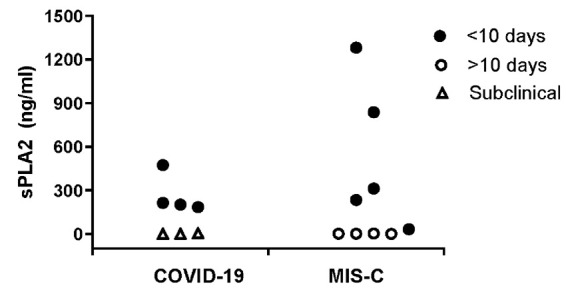
Secretory phospholipase A2 (sPLA2) levels in hospitalized children. Plasma sPLA2 levels (ng/mL) in symptomatic children diagnosed with acute COVID-19 infection (filled circles, *n* = 4) or multiorgan inflammatory syndrome in children (MIS-C) tested within 10 days of initiation of illness (filled circles, *N* = 5), subclinical children found to be RT-PCR positive for SARS-CoV-2 infection, screened due to hospitalization for other causes (unfilled triangle, *N* = 3) and those with MIS-C where blood sample was drawn during convalescences (>10 days after initiation of symptoms, with a range of 11–35 days, *N* = 4). Plasma sPLA2 levels are high in children with acute COVID-19 infection compared with a normal value <20 ng/mL, with highest sPLA2 levels identified in patients with MIS-C within 10 days of onset of symptoms. sPLA2 levels were significantly higher in patients with symptomatic COVID-19 infection or MIS-C within 10 days of onset of symptoms, compared with subclinical and convalescent patient samples (*P* = 0.01). A two-sided unpaired Student’s *t*-test was used to determine significant differences between acute and convalescent samples.

Patient demographics, laboratory values, and clinical course of the pediatric cohort are summarized in Table 2. A total of 24 children were assessed: 4 children hospitalized with symptomatic COVID-19 confirmed by PCR; 3 PCR+ children with subclinical infections who were hospitalized for other reasons but tested positive for SARS-CoV-2 by PCR (one patient with resolved upper respiratory symptoms who was admitted to the PICU for a suicide attempt, one patient with appendicitis who was tested prior to surgery, and one cardiac patient with presyncope who had no fever or respiratory symptoms); 9 children diagnosed with MIS-C, 3 children with KD not meeting the CDC criteria of MIS-C, and 5 additional hospitalized control subjects with fever, including 1 with Epstein-Barr virus–hemophagocytic lymphohistiocytosis (EBV-HLH; sPLA2 = 394 ng/mL), and 1 with Brucellosis (sPLA2 = 102 ng/mL); the latter 2 were not included in Table 2 analysis. Levels of sPLA2 varied widely in our pediatric cohort, but were significantly higher than values identified in the asymptomatic adult HCW cohort regardless of seropositivity status (188 ± 307, *n* = 24 vs. 9 ± 6, *n* = 28; *P* < 0.01); all pediatric versus adult participants, respectively. Overall, sPLA2 levels were elevated in children hospitalized with acute COVID-19, while the highest values were identified in children with acute MIS-C. Figure 1 illustrates sPLA2 levels in children with acute COVID-19 and MIS-C, differentiated by date of illness that the sample was obtained and by disease severity; all patients with either acute COVID-19 or acute MIS-C evaluated early in the course of illness (<10 days from the onset of symptoms; mean 6.2 ± 1.5 days, range 5–9 days) had significantly elevated sPLA2 levels compared with those with samples obtained later during the disease course (mean 26.5 ± 10.9 days after initiation of symptoms, range 11–35 days; *P* = 0.01). sPLA2 levels in patients with subclinical SARS-CoV-2 infection or in patients during convalescent phase of MIS-C (≥10 days after onset of symptoms) were not elevated. Children hospitalized with symptomatic acute COVID-19 had significantly elevated sPLA2 compared with those with subclinical disease (269 ± 137 vs. 2 ± 3 ng/mL, *P* = 0.01). Among children hospitalized with MIS-C, all had severe disease requiring PICU admission. However, sPLA2 levels were significantly higher in those in early (<10 days) versus late (≥10 days) disease (540 ± 510 vs. 2 ± 1, *P* = 0.04). White blood cell count (WBC) was elevated in patients with symptomatic COVID-19; however, the mean was skewed by two patients who presented simultaneously with new onset leukemia (WBC 176 and 118 × 10^3^ cells/µL). CRP and D-dimer levels were significantly elevated above normal in all categories (Table 2). There were no correlations identified between sPLA2 levels and WBC, CRP nor D-dimer, although a trend towards a correlation was identified between sPLA2 and CRP (*r* = 0.43, *P* = 0.06).

**Table 2.** Pediatric patient demographics, laboratory values, and clinical course.

Variables	All (*N* = 22)	COVID-19(*N* = 7)	MIS-C(*N* = 9)	Kawasaki(*N* = 3)	Fever^a^(*N* = 3)	*P* value^b^
Age, years, mean ± SD	9.9 ± 5.9	15.6 ± 5.8	8.8 ± 3.3	4.3 ± 3.2	5.7 ± 4.7	0.004
Gender: male, *N* (%)	13 (59%)	3 (43%)	5 (56%)	2 (67%)	3 (100%)	0.398
LOS (days), mean ± SD	14.0 ± 17.1	24.3 ± 27.2	12.0 ± 6.7	5.7 ± 2.1	4.0 ± 1.0	0.225
PICU: yes, *N* (%)	14 (64%)	4 (57%)	9 (100%)	1 (33%)	0 (0%)	0.008
Laboratory values^c^						
PCR+, *N* (%)	9 (41%)	7 (100%)	2 (22%)	0 (0%)	0 (0%)	0.001
IgG+, *N* (%)	9/14 (64%)	1 (50%)	8 (89%)	0 (0%)	0 (0%)	0.008
WBC^d^ ×10^3^ (cells/µL)	24.2 ± 42.2	59.1 ± 70.6	9.3 ± 4.7	14.7 ± 2.2	8.5 ± 6.3	0.004
CRP (µg/mL)	14.5 ± 8.7	12.0 ± 13.0	15.7 ± 7.9	19.3 ± 1.4	8.8 ± 2.3	0.448
D-dimers (ng/mL)	2401 ± 1679	1530 ± 530	3056 ± 1793	2792 ± 0	563 ± 393	0.204
sPLA2 (ng/mL)	190 ± 306	155 ± 172	301 ± 459	49 ± 67	33 ± 22	0.506

CRP: C-reactive protein; IgG: Immunoglobulin G; LOS: length of stay; MIS-C: multisystem inflammatory syndrome in children; PCR: polymerase chain reaction; PICU: pediatric intensive care unit; sPLA2: secretory phospholipase 2; WBC: white blood cell.

^a^Excluded febrile patients with Epstein-Barr virus/hemophagocytic lymphohistiocytosis and Brucellosis.

^b^One-way ANOVA for continuous variables or Chi-square test for categorical variables, alpha = 0.05. Data are represented as mean ± SD for continuous data or *N* (%) for nominal data.

^c^Denominators represent the number of patients in each group for whom testing was performed.

^d^Two patients with COVID-19 had new diagnoses acute myelogenous leukemia (AML) and highly elevated WBC.

## Discussion

This study provides preliminary data to suggest that children with acute COVID-19 and MIS-C have significantly elevated sPLA2 levels, with sPLA2 levels returning to normal during convalescence, supporting a potential role for sPLA2 in the COVID-19 inflammasome. This trend has been reported in other acute illnesses including sickle cell-ACS, pneumonia, acute asthma, and serious bacterial infections.^9,11,12,31^ Our anecdotal observation of very high sPLA2 levels in EBV-HLH is of interest given it is a syndrome of severe, life-threatening hyperinflammation; high levels in Brucellosis are also consistent with elevated sPLA2 in acute bacterial infections.^12^ sPLA2 may remain elevated in chronic inflammatory conditions like rheumatoid arthritis.^32^ However regardless of the trigger, elevated sPLA2 levels indicate a strong ongoing inflammatory signal and suggest the role of this enzyme in cell damage and organ failure.^2,5,8–11^ Normal values found in our convalescent adult HCWs also support the concept of normal sPLA2 levels during convalescence, following acute illness. Given a normal value for sPLA2 is <20 ng/mL,^12^ children with MIS-C evaluated within less than 10 days of illness demonstrated a 10–60 fold increase in sPLA2 levels. Normal levels observed in subclinical cases also support a link between sPLA2 levels and COVID-19 disease severity. Together, sPLA2 may represent an easily measured biomarker of COVID-19 and MIS-C that merits further evaluation in both children and adults. A recent report by Diorio *et al*. demonstrates an elevation in cytokine profiles associated with MIS-C,^33^ indicating that identifying inflammatory biomarkers associated with disease severity is valuable.

Despite early reports suggesting rare COVID-19 disease in children, more recent studies have found a considerable number of hospitalized and critically ill pediatric patients^34,35^ with many requiring pediatric intensive care unit (PICU) admission.^36^ Children with underlying medical conditions, including immune compromise and cardiorespiratory comorbidities appear to be at increased risk of severe COVID-19 disease.^37^ However, the newly described MIS-C associated with COVID-19 often affects previously healthy children with no underlying comorbidities.^38^ MIS-C is thought to be a postinfectious hyperinflammatory response to SARS-CoV-2 infection based on its temporal association with SARS-CoV-2, and detection of SARS-CoV-2 antibodies in affected children. MIS-C is defined by the CDC as 1) “an individual age < 21 years presenting with fever, laboratory evidence of inflammation, and evidence of clinically severe illness requiring hospitalization, with multisystem (≥2) organ involvement (cardiac, renal, respiratory, hematologic, gastrointestinal, dermatologic or neurologic); and 2) no alternative plausible diagnosis; and 3) positive for current or recent SARS-CoV-2 infection by RT-PCR, serology or antigen test; or exposure to a suspected or confirmed COVID-19 case within the 4 weeks prior to the onset of symptoms.”^39^

Children with MIS-C have high rates of PICU admission, mechanical ventilation, and vasopressor requirements.^40,41^ Complications include myocarditis, cardiorespiratory failure, and even death.^42^ The emergence of MIS-C as a novel and serious pediatric condition highlights the importance of studying the impact of SARS-CoV-2 infection in children as well as adults. Ultimately, a biomarker of pediatric COVID-19 and MIS-C disease severity would be highly valuable to stratify risk and to guide patient management.

**Figure 2.**
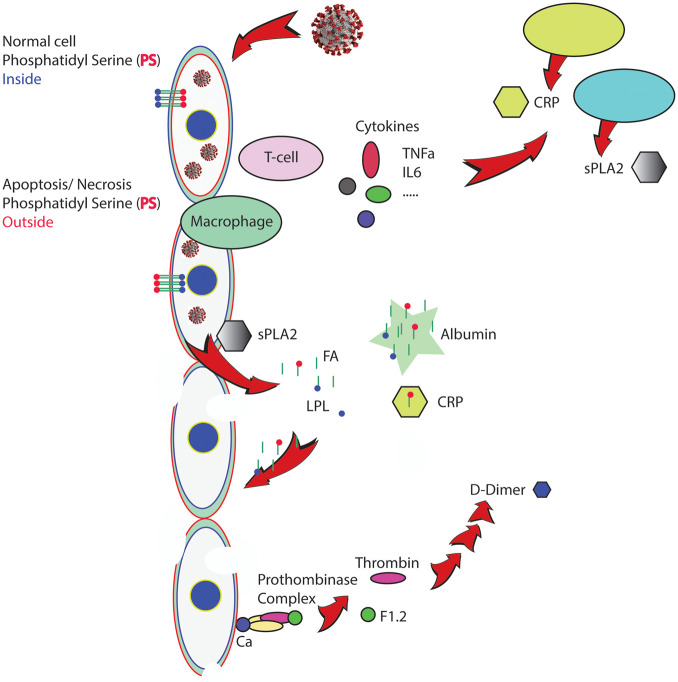
Secretory phospholipase A2 (sPLA2)-related COVID-19 inflammasome. Simplified scheme of processes that will lead to vascular damage and multiorgan failure in COVID-19 patients. Cytokines initiate the production of sPLA2. Invasion by the virus renders the cell apoptotic, a process that activates phosphatidylserine (PS) exposure, a signal for cell removal. Overwhelming numbers of PS exposing cells are targets for sPLA2, generating lysophospholipids and fatty acids, powerful detergents that will damage additional cells if not properly buffered. PS exposure will also activate the prothrombinase complex, starting the coagulation process.

New evidence is emerging daily that COVID-19 is more than a respiratory disease; it can also result in multiorgan failure and coagulopathy in severely ill patients. While viremia obviously triggers the pathology observed, clinical data suggest that the immune system plays an important role in the morbidity and mortality of COVID-19. This in turn has generated interest in treatments such as corticosteroids and immunomodulatory agents that mitigate the immune response. However, treatments that act broadly to suppress the immune system have the potential to impede the body’s ability to control the viral infection. While corticosteroids reduce the formation of sPLA2 and may benefit severe cases of COVID-19,^43–47^ their use has also been related to adverse events,^48^ as shown among relatively healthy recipients in a large study from Taiwan.^49^ More focused treatments that address specific inflammatory pathways, including blocking the effect of specific inflammatory factors may be advantageous. Since levels of sPLA2 in blood of pediatric patients appear to correlate with severity, we propose that elevated levels of sPLA2, together with apoptotic changes in cellular membranes, lead to vascular dysfunction. Apoptosis is a normal and continuous process in tissue remodeling. A primary signal that makes the cell recognizable as apoptotic is the loss of phospholipid asymmetry and exposure of PS on its surface. Apoptotic cells are removed in a highly orchestrated way by macrophages, before the cell membrane viability is lost and the cell will lose its content. Viral infection leads to apoptosis, PS exposure, and is related to the macrophage removal of virus-infected cells.^50–54^ Endothelial cell infection and endothelitis have been reported in COVID-19.^55^ Whereas this report focused on ACE2 receptors expressed by endothelial cells, the data showed apoptosis of endothelial cells and mononuclear cells. Similarly, as observed for lung damage in SCD patients during ACS, endothelial damage and vascular dysfunction in COVID-19 will affect the vascular health in all organs including the brain. Figure 2 shows a working model to illustrate our proposed link between the COVID-19 inflammasome, upregulated sPLA2 levels, and cellular damage. Clinical severity of COVID-19 appears related to the degree of viremia, and it is logical to assume that the number of apoptotic cells formed is also related to the viral dose. We hypothesized that under these conditions PS exposing cells are not efficiently removed, and sPLA2 will break down cells that expose PS.^8^ Lipolysis of these damaged cells by sPLA2 will generate non-esterified fatty acids (NEFA) and lysophospholipids (LPLs) and lead to release of cellular content in the environment. Increased levels of NEFA and LPL were found in plasma of sickle cell patients diagnosed with ACS.^56^ These sPLA2-induced cellular breakdown products will affect other cells in the circulation when not properly buffered or removed. CRP, similarly upregulated by inflammatory cytokines as sPLA2, provides a binding site for LPL, and we found a clear correlation between levels of CRP and sPLA2 in patients with SCD that develop ACS.^57^ Recent data have shown a possible correlation between levels of CRP and severity in subsets of COVID-19 patients.^58–60^ However, the correlation between upregulated CRP and severity is not always clear, and extensive formation of LPL and FA will overwhelm the normal buffering by CRP as well as albumin or lipoproteins.

Our findings that link sPLA2 to clinical disease severity provide a rationale for therapies to stabilize the endothelium while tackling viral replication, including specific anti-inflammatory drugs, specific inhibitors of sPLA2 formation or activity, as well as compounds that “cloak” its target, PS exposing cells. Compounds that affect the formation of sPLA2 include IL-6 inhibitors such as tocilizumab, as well as potential IL-6 blockers including sarilumab, ALX-0061, sirukumab, MEDI5117, clazakizumab, and olokizumab.^61^ Infliximab, a chimeric monoclonal TNF-alpha antibody, is used to treat a number of autoimmune diseases including Crohn's disease, ulcerative colitis, rheumatoid arthritis, ankylosing spondylitis, psoriasis, psoriatic arthritis, and Behçet's disease. Its action has been related to a decrease in the formation of sPLA2.^62^ Compounds like Varespladib, a sPLA2 inhibitor,^63^ and other specific inhibitors of sPLA2 may warrant further investigation as therapeutic agents in COVID-19 and MIS-C. The use of a sPLA2 inhibitor in sepsis demonstrated improved survival in a subgroup of patients who received the drug within 24 h of sepsis-induced organ failure. Results in a larger group with severe organ failure were not significant,^64^ confirming that the administration of these compounds is needed before major organ damage has occurred.

As has been demonstrated with corticosteroids, modifying the inflammatory response carries a risk. However, lowering cellular damage invoked by the attack of sPLA2 may provide an additional tool to address unchecked inflammation. Cloaking PS surfaces may also represent a strategy to mitigate the prothrombotic state observed in COVID-19 patients. Unwanted presence of PS exposing cells will dysregulate hemostasis. The assembly of the prothrombinase complex on the PS exposing surface of activated platelets, and formation of thrombin and F1.2 starts the coagulation process. PS exposure on other cells will lead to a prothrombotic state, and PS exposing sickle-erythrocytes are related to F1.2 levels in plasma. Early reports of abnormal coagulation parameters in COVID-19 patients from Wuhan^65^ were confirmed with additional studies.^66,67^ Elevation of D-dimer and thrombus formation was reported in COVID19,^68^ and low molecular weight heparin treatment appeared to associate with outcomes.^69^ We suggest therefore that “cloaking” of the PS exposing surface may lower both sPLA2-induced damage as well as the formation of thrombin.

Annexin is a common name for a group of cellular proteins that bind to PS exposing membranes in the presence of calcium. As a fluorescent derivative annexin is widely used to visualize apoptotic cells by microscopy or flow cytometry.^70^ We developed di-annexin as a compound with a longer lifetime in the circulation to “cloak” PS exposing surfaces.^71^ This compound has proven to be effective in modulation ischemia reperfusion injury in animals,^72–77^ and has been used in solid organ transplants.^74,78,79^ We speculate that this compound could lower both the damage invoked by sPLA2 as well as the onset of thrombotic events.

Ultimately, treatment options for COVID-19 will rely on a combination of the inhibition of viral replication, anticytokine, and anti-inflammatory agents. We additionally propose an approach that specifically target sPLA2 and PS exposing cells.

Our small sample size of children with acute COVID-19 and MIS-C at a single center is a limitation, and the data may not be generalizable. Future work with a larger sample size and multicenter collaboration is necessary to broaden our understanding of the virus and its consequences in children. However, our data provide a proof-of-concept regarding a correlate of sPLA2 in COVID-19 and MIS-C, justifying further investigation. Variable timing of blood sampling is another limitation; measurements earlier in the course of illness and longitudinal analysis of sPLA2 would provide further insight. In SCD, for instance, daily assessment of sPLA2 levels predicted the onset of ACS.^9,10,31^ Additionally, we did not control for anti-inflammatory interventions that may have impacted sPLA2 levels. Future studies will be necessary to further understand how this enzyme correlates with COVID-19 disease severity and pathogenesis in adults. Data from our asymptomatic HCW cohort confirm that sPLA2 is normal in adults with a history of COVID-19 in convalescence; however, sPLA2 levels were not available during acute illness, and none of our adult participants qualified as having had “severe” disease based on the need for inpatient hospital admission, in contrast to our hospitalized pediatric cohort.

Together, our data indicate that high levels of sPLA2 predict clinical disease severity in children, both in the acute phase of COVID-19 and in those who develop MIS-C. These results can inform hypotheses for future studies. The assay to measure sPLA2 can easily be introduced as a routine assay in the acute care setting, added to the measurements provided by a clinical lab. The sPLA2 results may provide a tool for the clinician to decide on a course of action to benefit the patient, and may avoid delayed diagnosis of COVID-19-related pathology.^80^ We suggest that therapeutic compounds targeting sPLA2, as well as those specifically aimed at lowering vascular damage of PS exposing cells, warrant consideration.
